# Highly competitive fungi manipulate bacterial communities in decomposing beech wood (*Fagus sylvatica*)

**DOI:** 10.1093/femsec/fiy225

**Published:** 2018-11-29

**Authors:** Sarah R Johnston, Jennifer Hiscox, Melanie Savoury, Lynne Boddy, Andrew J Weightman

**Affiliations:** Cardiff School of Biosciences, Cardiff University, Museum Avenue, Cardiff. CF10 3AX. Wales, UK

**Keywords:** fungi, bacteria, decomposition, wood, succession, pH

## Abstract

The bacterial communities in decomposing wood are receiving increased attention, but their interactions with wood-decay fungi are poorly understood. This is the first field study to test the hypothesis that fungi are responsible for driving bacterial communities in beech wood (*Fagus sylvatica*). A meta-genetic approach was used to characterise bacterial and fungal communities in wood that had been laboratory-colonised with known wood-decay fungi, and left for a year at six woodland sites. *Alpha*-, *Beta*- and *Gammaproteobacteria* and *Acidobacteria* were the proportionally dominant bacterial taxa, as in previous studies. Pre-colonising wood with decay fungi had a clear effect on the bacterial community, apparently via direct fungal influence; the bacterial and fungal communities present at the time of collection explained nearly 60% of their mutual covariance. Site was less important than fungal influence in determining bacterial communities, but the effects of pre-colonisation were more pronounced at some sites than at others. Wood pH was also a strong bacterial predictor, but was itself under considerable fungal influence. *Burkholderiaceae* and *Acidobacteriaceae* showed directional responses against the trend of the bacterial community as a whole.

## Teaser

The identity of the bacteria living in wood is influenced by the identity of the fungi living there.

## INTRODUCTION

Wood-decay fungi are the major terrestrial agents of wood decomposition, known for their highly territorial and competitive ecological strategies (Boddy 2000; Boddy *et al*. 2017). Despite the extensive literature on inter-fungal interactions, little is known about how they interact with bacteria in wood (de Boer *et al*. 2005; Johnston, Boddy and Weightman 2016). The interactions between fungi and bacteria in dead wood are likely to have ramifications for ecosystem processes, particularly the rate of wood decomposition. Given that different fungi decompose wood at very different rates, anything that affects fungal community composition will have knock-on impacts on the regulation of wood decay and release of the nutrients within (Crowther, Boddy and Jones 2011). Although direct bacterial contributions to decomposition are likely to be modest, bacteria could have an indirect effect on decomposition rates by consuming the breakdown products of fungal enzymatic activity, thus preventing enzyme down-regulation via feedback inhibition (de Boer *et al*. 2005; Johnston, Boddy and Weightman 2016).

With the rise of high-throughput sequencing, accurate bacterial surveys have become possible: the culturable fraction of dead-wood-inhabiting bacteria can be as low as 1% (Folman *et al*. 2008). Surveys so far indicate a diverse suite of bacteria within decomposing wood (Zhang, Yang and Tu 2008; Hoppe *et al*. 2014, 2015; Sun *et al*. 2014; Rinta-Kanto *et al*. 2016; Kielak *et al*. 2016b; Tláskal *et al*. 2017). Many physical properties of wood, such as pH, decay state, moisture content and C:N ratio influence bacterial community composition (Hoppe *et al*. 2015; Tláskal *et al*. 2017). Each of these can be altered by fungal activity. Microcosm studies already indicate that wood-decay fungi modify bacterial communities within a woody resource (Folman *et al*. 2008; Hervé *et al*. 2014), and there is evidence that certain bacterial and fungal taxa associate non-randomly in the field (Hoppe *et al*. 2014; Kielak *et al*. 2016b).

Bacterial diversity in wood is highly heterogeneous within and between sites (Sun *et al*. 2014; Hoppe *et al*. 2015). Soil type (Sun *et al*. 2014) and the surrounding forest management regime (Hoppe *et al*. 2015) are important predictors of inter-site variability. At the landscape scale, pH is the major driver of soil bacterial communities, and bacterial richness increases with pH (Fierer and Jackson 2006; Griffiths *et al*. 2011). If soil is the primary source of wood-inhabiting bacteria, then local soil pH would be expected to constrain the pool of potential colonists. Bacteria entering the wood would undergo a second round of selection by pH, as wood usually represents an acidic environment and many wood-decay fungi dramatically lower the surrounding pH (de Boer *et al*. 2010). Under this scenario, it would be expected that bacterial diversity in wood would be negatively correlated with wood pH, and that this effect would be most obvious at sites with a high soil pH (where there is a more diverse pool of colonists).

Fungal decomposition of wood is a dynamic process, carried out by a successional series of fungi. This means that, at any given time, the properties of a given resource are determined not only by its current community, but also by its history. Each fungus has a particular chemical signature of decomposition (Schilling *et al*. 2015), and also varies idiosyncratically in its ability to hold territory against invaders (Boddy 2000; Boddy *et al*. 2017; Hiscox *et al*. 2017). These factors lead to priority effects in wood: distinctive patterns of successor species dictated by the identity of former colonists (Hiscox *et al*. 2015, 2016). In light of this dynamic succession, it may be more meaningful to think of bacteria associating with a particular fungal community, rather than a particular fungus, with that community shaped by both the currently dominant fungus, and a succession of predecessors. It is also possible that these priority effects operate on bacteria directly, due to the biochemical legacy of fungi that have been replaced.

This study used a manipulative field experiment to characterise the bacterial community in decomposing wood at six UK woodland sites, with explicit reference to the fungi present. It tests three predictions: (1) that the bacterial community would vary depending on the identity of the original fungal colonist; (2) that the bacterial community would be significantly correlated with the identity of the fungal community present at time of sampling; and (3) that there would be inter-site differences in the bacterial community. Subsequent to testing these formal hypotheses, exploratory analysis was conducted to further characterise the drivers of bacterial community composition.

## MATERIALS AND METHODS

### Overview

Wood disks were lab-colonised with wood-decay fungi, and exposed for 1 yr on the floor at six woodland sites across the southern UK. After collection, bacterial and fungal communities were characterised by amplicon sequencing. The fungal analysis has been published elsewhere (Hiscox *et al*. 2016), and is only dealt with here insofar as it pertains to the bacterial data.

### Field experiment

Branches from beech trees (*Fagus sylvatica*) were felled and cut into sections approx. 2 cm thick and 10–20 cm in diameter. Wood disks were frozen after cutting, and sterilised by autoclaving three times in a 72-hr period. Disks were colonised for 3 months by single wood-decay fungi (Table 1) on 0.5% malt agar (5 g l^−1^ malt, 15 g l^−1^ agar no. 2, LabM, UK) in 400 ml plastic tubs (Cater4you, UK). Twenty-five percent of disks were kept sterile and frozen at −20°C as uncolonised controls. In autumn 2012, surface mycelium was scraped off, and disks colonised by each of the three fungal species, with uncolonised controls, (nine replicates) were placed on the forest floor at random positions on a grid at each of six sites across the southern UK (Table S1). All field sites were mixed deciduous woodland containing predominantly *F. sylvatica*.

**Table 1.** Fungal species used to colonise disks. All fungi are white-rot wood-decay basidiomycetes from the Cardiff Culture Collection.

Name	Strain	Family	Ecological strategy	Competitive ability	Acronym
*Hypholoma fasiculare*	HfDD3	Strophariaceae	Late stage secondary/ tertiary colonist; cord former	High	Hf
*Trametes versicolor*	TvCCJH1	Polyporaceae	Early-mid stage secondary colonist	Intermediate	Tv
*Vuilleminia comedens*	VcWVJH1	Corticiaceae	Primary colonist	Low	Vc

Disks were collected after 1 yr (autumn 2013) and transported back to the lab individually in sealed plastic bags. A soil sample was taken directly below each disk for pH analysis. Each disk was surface-sterilised with 10% household bleach, before drilling with a sterile drill bit at random points spaced evenly across the face of the disk. Wood dust (swarf) was flash-frozen in liquid N_2_ and stored at −80°C. At six points on each face of the disk, chips of wood were removed aseptically and re-isolated onto 2% malt agar to assess pre-coloniser persistence (via mycelial morphology and somatic incompatibility). DNA was extracted from 0.3 g swarf using the MoBio PowerSoil® kit (Carlsbad, CA USA), replacing the vortex step with 3 × 20 s bead-beating at 4 m s^−1^ in a MP FastPrep®-24. For each disk, 0.5 g from a second aliquot of swarf was added to 5 ml distilled water and mechanically shaken for 1 h; pH was measured using a Hanna Instruments pH20 pH meter. Soil pH readings were taken by the same method.

### Molecular analysis

Joint fungal–bacterial community analysis was performed on four disks per treatment per site, with the exception of disks pre-colonised with *H. fasciculare* at Bagley. In this case, replicates were lost during the field exposure (probably due to mammal activity), and the treatment had to be excluded from the analysis.

The fungal ITS2 region was amplified using gITS7 (GTGARTCATCGARTCTTTG) and ITS4 (CCTCCGCTTATTGATATGC) (Ihrmark *et al*. 2012; Hiscox *et al*. 2016). PCRs were carried out in 50 µl reactions (2.5 µl template, 300 nM tagged ITS4, 500 nM gITS7, 0.025 U HS Taq polymerase (PCRBiosystems, UK), 10 µl supplied buffer) in a Dyad DNA Engine Peltier thermal cycler. The initial incubation was 5 min at 94°C; followed by 22–30 × (30 s at 94°C; 30 s at 56°C; 30 s at 72°C) and 7 min at 72°C. Triplicate PCRs per sample were pooled equimolarly based on image analysis using ImageJ software (Rasband, 1997–2014), purified with the QIAQuick gel extraction kit (Qiagen, Germany), and quantified with the Quant-iT PicoGreen dsDNA assay kit (Life Technologies Ltd, UK). Samples were sequenced on a Roche 454 GS FLX+ (Hoffman La-Roche Ltd., Germany) by the NERC Biomolecular Analysis Facility, Centre for Genomic Research, Liverpool, UK.

PCR and sequencing of the bacterial 16S rRNA gene region from the same samples was carried out by the Institute of Applied Biotechnologies, Prague, Czech Republic. Triplicate PCRs were performed using primers S-D-Bact-0341-b-S-17 (CCTACGGGNGGCWGCAG) and S-D-Bact-0785-a-A-21 (GACTACHVGGGTATCTAATCC) (Klindworth *et al*. 2013), and the products pooled. This primer pair targets the V3-V4 region of the 16S rRNA gene, and shows excellent taxonomic coverage (Klindworth *et al*. 2013). Samples were sequenced on an Illumina MiSeq (v3, 2 × 300 base-pair reads) (Illumina, Inc., San Diego, USA) with Nextera XT assay chemistry.

### Sequence analysis

Fungal sequence data were processed as described by Hiscox *et al*. (Hiscox *et al*. 2016). For bacterial sequences, paired-end reads were joined and demultiplexed by the sequencing provider. Detailed commands and parameters used during bioinformatic processing are given in supplementary material. Sequences were filtered using a custom script to retain only those with complete, error-free primer regions, and the primers and barcodes were removed. USEARCH v9.0.2132 (Edgar 2010) was used to exclude sequences with <400 base pairs or >2 expected errors, before downstream analysis with QIIME 1.9.1+dfsg-1biolinux4 (Caporaso *et al*. 2010). Chimeric sequences were identified in QIIME using USEARCH 61 and removed. Sequences were clustered into operational taxonomic units (OTUs) by open reference picking in order to balance breadth of coverage and computation time, using the Greengenes 16S rRNA gene database (DeSantis *et al*. 2006) at 97% sequence similarity. Singletons (OTUs occurring only once) were removed at this stage. To check for fungal sequence contamination, OTU picking was repeated against the SILVA_119 16S/18S rRNA gene database (Pruesse *et al*. 2007; Quast *et al*. 2013). No sequences were assigned to fungi, so the Greengenes OTUs were used for subsequent analysis. Relative abundance plots were produced in QIIME with *summarize_taxa_through_plots.py*. Fungal sequence data are archived at NCBI SRA (accession number SRP052547) and bacterial sequence data at the European Nucleotide Archive (ENA) under accession number PRJEB22364.

### Statistical analysis

Analysis was performed in R (R Development Core Team 2011) using RStudio (RStudio Team 2016) and packages *dplyr* (Wickham and Francois 2016), *ggplot2* (Wickham 2009), *metacoder* (Foster 2016) and *vegan* (Oksanen *et al*. 2016). All R code to reproduce the analyses is available as an R markdown file at *github.com/ecologysarah/fungi-bacteria-multisite* (Allaire *et al*. 2016). Test statistics and significance values were calculated for formal hypothesis tests. For the exploratory analysis, patterns in the data were quantitatively characterised but significance values are not provided, as they become meaningless and inappropriate in the absence of an *a priori* hypothesis (Nuzzo 2014).

The nature of sequencing technology means that raw amplicon data vary hugely in sequence numbers (creating unequal sample sizes); the present bacterial dataset ranged from 866 – 70 814 reads per sample (median 27 900). This is normally dealt with by rarefying (randomly subsampling data to an equal numbers of observations). The practice has come under valid criticism, but lacks robust alternatives (McMurdie and Holmes 2014; Weiss *et al*. 2017). After careful data exploration, it was decided not to rarefy the current dataset, for the following reasons: (a) Sequencing depth did not co-vary with any of the factors of interest, *i.e*. depth is an unwanted variable randomly distributed across treatments. (b) NMDS of the bacterial data revealed that variation due to sequencing depth could be separated out on a single axis, independently of other predictors, thus allowing visualisation independent of depth. (c) For OTU richness assessment, modelling and residual-based analysis provide a more robust and explicit way to deal with sequencing depth. (d) Where abundances were required for plotting or Procrustes analysis, proportions were used in place of raw read counts. Amplicon data are inherently compositional, so proportions simply scale the data to make them comparable (Lovell *et al*. 2010).

### Community analysis

Permutation ANOVA (PERMANOVA; 999 permutations) was used on a Bray–Curtis distance matrix for formal significance testing of pre-coloniser and site effects (Anderson 2001). Sequencing depth was included as a continuous predictor. Owing to the lack of *post hoc* tests for PERMANOVA, the dataset was then broken down into pairwise combinations and PERMANOVA run separately on each (Hiscox *et al*. 2016). Pairwise tests were conducted for species differences between sites (not site differences between species) to limit the number of tests. *P*-values for the pairwise tests were subjected to the Benjamini–Hochberg false discovery rate (FDR) correction for multiple testing (Benjamini and Hochberg 1995). To relate whole bacterial and fungal communities to each other, Procrustes analysis was used to superimpose the two unrarefied OTU tables (999 permutations). Subsequently, the Procrustean association metric (PAM) was extracted and regressed individually against pre-coloniser, site, wood pH and soil pH using one-way ANOVAs (Lisboa *et al*. 2014). The response variable was natural log-transformed to meet parametric assumptions.

Bacterial community composition between samples was visualised using non-metric dimensional scaling (NMDS) on a Bray–Curtis distance matrix. Given that overdispersion in the data can introduce artefacts in distance metrics (Warton, Wright and Wang 2012), the ordination was validated by qualitative comparison with principal components analysis using a Hellinger transformation (Fig. S1, Supporting Information). To compare community composition simultaneously at multiple taxonomic levels, heat trees were plotted for the different treatments (Foster, Sharpton and Grünwald 2017). The taxonomic composition of the samples is displayed in a tree format, with node size and colour dictated by the relative abundance of each taxon.

### Richness

OTU richness was strongly correlated with sequencing depth in the raw data. To correct this without discarding data was by rarefying, a linear model was run with sequencing depth as the sole predictor. The response variable was natural log-transformed to meet parametric assumptions. Back-transformed residuals were calculated and plotted instead of the raw data, to control for sequence depth. To further characterise drivers of richness, a general linear model was run with sequencing depth, pre-coloniser, site, wood pH and soil pH as predictors. The response variable was again *ln*-transformed. Coefficients from the model were extracted and back-transformed to quantify the relative importance of the different predictors.

### Exploration of selected taxa

OTUs from three bacterial families (*Acetobacteraceae*, *Acidobacteriaceae* and *Burkholderiaceae*) were selected for further exploration due to their apparent association with fungal pre-colonisers, based on graphical patterns. OTU richness within each of these families was modelled by the same process as for overall richness (see above), with the exception that no data transformation was necessary.

### Relationships between predictors

The relationships between abiotic factors and the fungal community were explored graphically. Because wood-decay fungi are known to manipulate pH, one-way ANOVAs were used to assess how much of the variation in wood pH could be attributed to past or current fungal activity.

## RESULTS

### Preliminary analysis of sequencing data

Of the bacterial paired-end reads,  2 710  316 passed quality filtering and were grouped into 7 380 OTUs. One *T. versicolor* pre-colonised disk from Tintern was excluded from subsequent analysis due to concerns that it had been mislabelled.

### Relationships between predictors

Soil pH varied strongly between sites. Wood pH varied to a lesser extent between pre-colonisers and sites, but there was no relationship between wood pH and soil pH (Fig. S2, Supporting Information). Pre-coloniser identity explained 8.5% of the variation in wood pH, whereas the genus of the dominant fungal OTU at time of sampling explained 65.5% (adjusted *R^2^*, one-way ANOVA).

### Pre-coloniser and site effects on bacterial community composition

**Figure 1.**
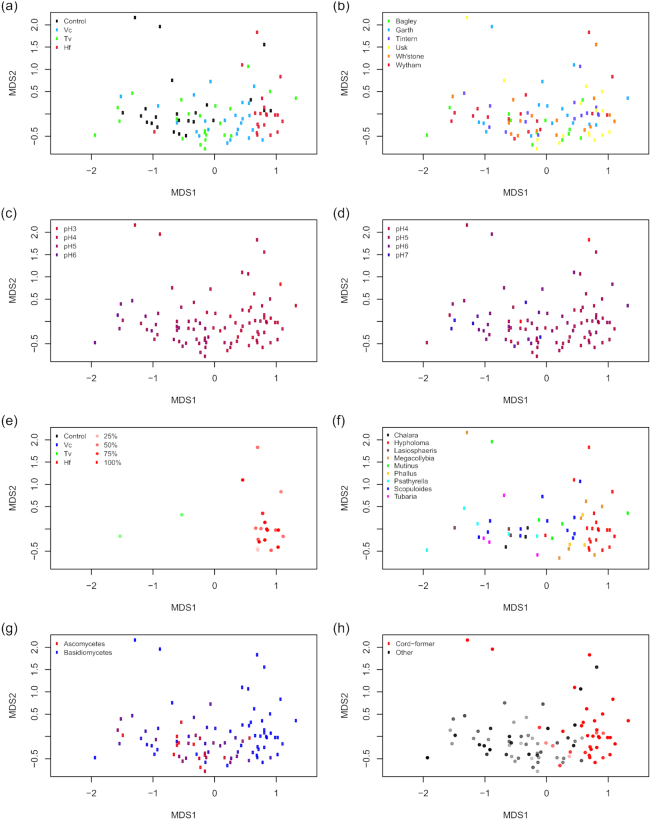
NMDS ordination of the bacterial community in fungus-colonised wood disks. Points are coloured by (**a**) pre-coloniser identity; (**b**) site of origin; (**c**) gradient of wood pH, with high to low pH represented by colour gradient; (**d**) gradient of soil pH likewise; (**e**) pre-coloniser persistence (colour dictated by identity, transparency by persistence i.e. the percentage of reisolation points from which the pre-coloniser was isolated); (**f**) genus of the dominant fungal OTU (only genera with three or more records are shown); (**g**) relative proportions of ascomycete and basidiomycete reads and (**h**) whether the dominant fungal OTU belonged to a cord-forming genus (transparency dictated by % of disk held by the dominant OTU). Colour gradients correspond to the squared pH values in order to improve visual colour differentiation. Stress = 0.148

Axis 1 of the NMDS separated samples by pre-coloniser (specifically, *H. fasciculare* samples clustered separately to other treatments (Fig. 1a). The second axis was explained by sequencing depth (Fig. S3, Supporting Information). There was little patterning by site, other than limited clustering of samples from the Usk site (Fig. 1b). Neither soil pH nor wood pH showed a clear pattern in the NMDS (Fig. 1c and d).

The overall PERMANOVA showed a highly significant interaction between pre-coloniser and site (F_14,67_ = 1.807, *P* = 0.001). The pairwise tests revealed significant differences between all treatments at the Wytham site (Table 2, Fig. S4, Supporting Information).

**Table 2.** Adjusted *P*-values from pairwise PERMANOVA comparisons of bacteria community composition.

Comparison	Bagley	Garth	Tintern	Usk	Whitestone	Wytham
Control-Vc	**0.014**	**0.033**	0.471	0.098	**0.024**	**0.005**
Control-Tv	0.103	**0.042**	***0.013***	0.103	**0.007**	**0.022**
Control-Hf	–	**0.016**	**0.013**	**0.010**	**0.005**	**0.014**
Vc-Tv	***0.014***	0.185	0.110	**0.007**	0.501	**0.005**
Vc-Hf	–	0.110	**0.028**	***0.005***	0.366	**0.005**
Tv-Hf	–	0.103	**0.012**	***0.005***	0.125	**0.005**

All numbers are given to three decimal places. Values in italics should be regarded with caution, as the between-group dispersions were unequal for those subsets of the data. Significant values are given in **bold**.

**Figure 2.**
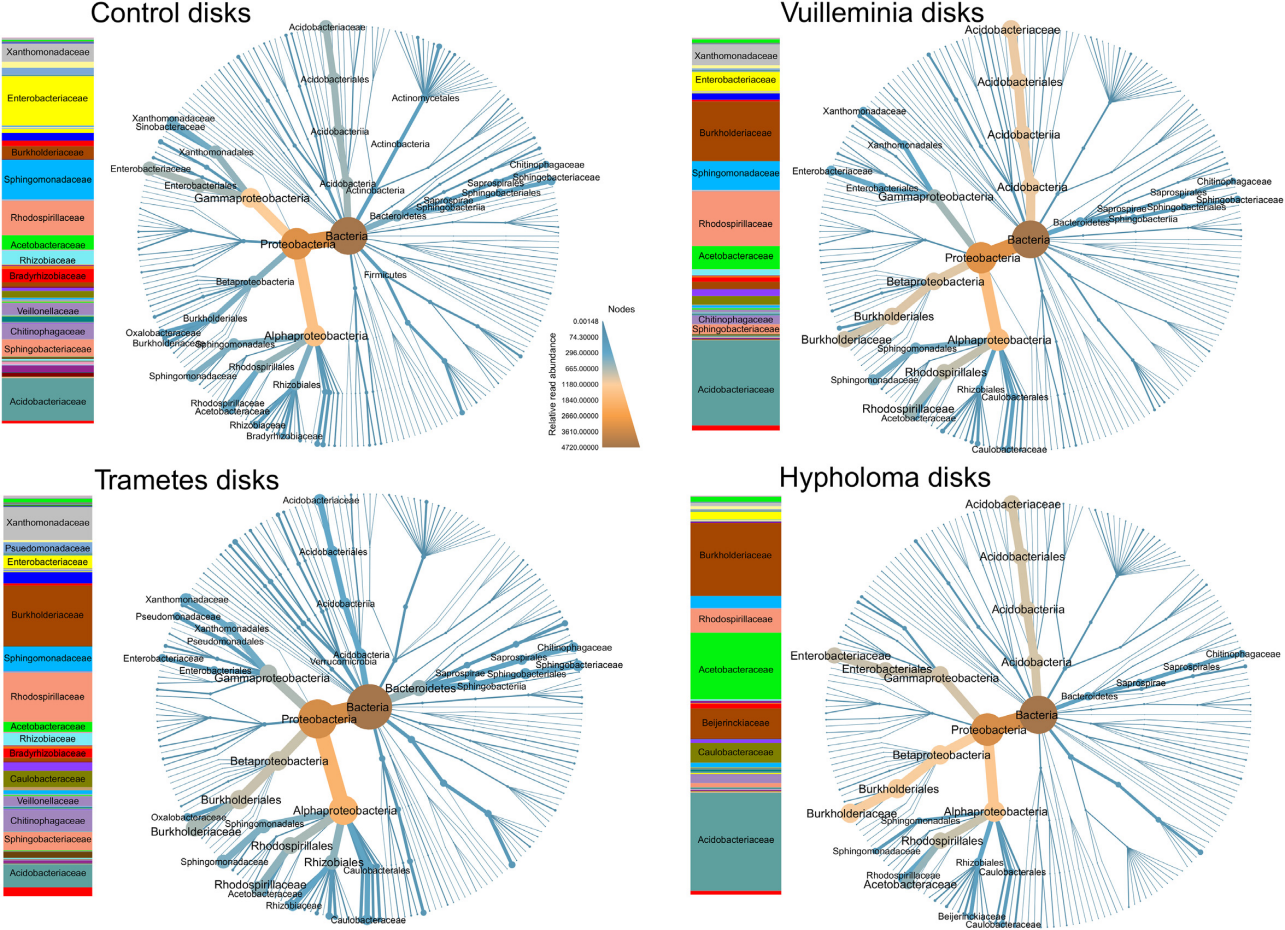
Taxonomic composition of the bacterial community in fungus-colonised wood disks, broken down by treatment. Stacked bar charts of family relative abundance are presented alongside heat trees. Node colour and size on the heat trees represent relative abundance for that taxon; relative read abundance is on an arbitrary scale where OTUS within each sample sums to 100 (taxa may score higher than 100 as they aggregate multiple samples). Reads unassigned at the domain level are excluded for ease of visualisation.

*Proteobacteria* were dominant in all treatments, *Alphaproteobacteria* consistently so with *Betaproteobacteria* and *Gammaproteobacteria* more variable among treatments (Fig. 2). Both *V. comedens* and *H. fasciculare* disks showed enrichment in *Acetobacteraceae* and *Acidobacteriaceae* (Fig. 2; Table 3). All three pre-coloniser treatments were enriched in *Burkholderiaceae*, and showed a decrease in *Actinobacteria* and *Gammaproteobacteria*. Also noteworthy was the prominence of *Chitinophagaceae* in all treatments. A number of taxa also showed differences in relative abundance between sites (Fig. S5, Supporting Information): Bagley had proportionately more *Acidobacteria* and fewer *Gammaproteobacteria* than the other sites, whilst Garth was the only site where *Firmicutes* were prominent. Most sites showed reciprocal abundance between *Enterobacteriaceae* and *Xanthomonadaceae*.

**Table 3.** Estimates from general linear models on OTU richness.

Coefficient	Overall richness	*Burkholderiaceae*	*Acidobacteriaceae*	*Acetobacteraceae*
Control-Vc	0.896‡	12.8	9.34	0.754
Control-Tv	0.831‡	14.3	-4.67	−2.61
Control-Hf	0.776‡	13.6	10.5	3.85
Vc-Tv	0.928‡	1.44	-14.0	−3.36
Vc-Hf	0.866‡	0.814	1.21	3.10
Tv-Hf	0.933‡	−0.627	15.2	6.46
Bagley–Garth	0.933‡	−17.7	−0.725	7.16
Bagley–Tintern	1.118‡	−10.7	3.52	6.04
Bagley–Usk	0.801‡	−10.4	−2.73	4.66
Bagley–Whitestone	1.0978‡	−15.1	−3.19	3.74
Bagley–Wytham	0.967‡	−9.89	4.03	8.01
Garth–Tintern	1.199‡	7.00	4.24	−1.12
Garth–Usk	0.859‡	7.27	−2.01	−2.51
Garth–Whitestone	1.177‡	2.52	−2.47	−3.42
Garth–Wytham	1.0371‡	7.75	4.76	0.846
Tintern–Usk	0.716‡	0.275	−6.25	−1.39
Tintern–Whitestone	0.982‡	−4.48	6.71	−2.30
Tintern–Wytham	0.865‡	0.758	0.516	1.97
Usk–Whitestone	1.371‡	−4.75	−0.462	−0.911
Usk–Wytham	1.208‡	0.483	6.76	3.35
Whitestone–Wytham	0.881‡	5.23	7.23	4.27
Wood pH	1.395‡	−16.8	−11.3	−4.52
Soil pH	1.0981‡	−3.60	−5.67	−4.51
Sequencing depth	1.0000176‡	0.000526	0.000495	0.000312

For the overall richness model, estimates were obtained by backtransforming coefficients from the model (marked ‡). Values represent the ratios of geometric means when moving between levels (categorical predictors, i.e. Vc has 89.6% richness of the control) or for a one unit increase in the predictor (continuous predictors, i.e. a one-unit increase in wood pH corresponds to a 39.5% increase in OTUs). The individual family models did not require transformation, so each estimate simply represents the average increase in OTU numbers between levels (categorical predictors) or the average increase in OTU numbers for a one-unit increase in the predictor (continuous predictors). All numbers are given to three significant figures. Abbreviations are control (C), *Vulleminia comedens* (Vc), *Trametes versicolor* (Tv), *Hypholoma fasciculare* (Hf).

### Fungal community effects on bacterial community composition

Of the fungal pre-colonisers, only *H. fasciculare* could still consistently be re-isolated after 1 yr (Fig. 1e). A diverse range of fungi colonised the disks; considering just the dominant OTU within each disk (i.e. the OTU that accounted for a simple majority of reads), 18 identifiable genera were represented, of which nine were dominant in three or more disks each (Fig. 1f). The relative proportion of ascomycetes versus basidiomycetes within each disk did not produce a discernible pattern in the bacterial community (Fig. 1g). The clearest separation in bacterial communities arose between samples with a dominant fungal OTU belonging to a genus of known cord-formers (fungi that disperse via thickened cords of mycelium, marked out as a group by their highly competitive ecological strategies; Boddy 1993), compared to those where the dominant OTU was not a known cord-forming species (L. Boddy, unpubl. data) (Fig. 1h).

**Figure 3.**
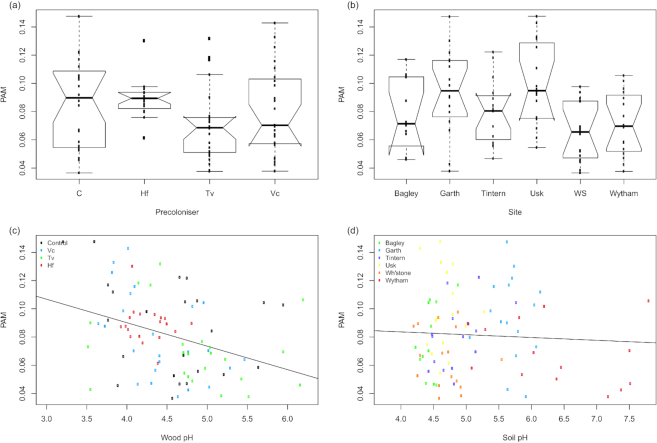
Procustean association metric (PAM) between the fungal and bacterial communities. PAM shows the residual variation plotted against (**a**) pre-coloniser identity; (**b**) site of origin; (**c**) wood pH (coloured by pre-coloniser) and (**d**) soil pH (coloured by site). Individual data points are overlaid on boxplots. A simple regression line is overlaid on the scatterplots. Notches on boxplots represent 95% confidence intervals; where these extend beyond the quartiles, ‘hinges’ appear on the plot. Abbreviations are control (C), *Vulleminia comedens* (Vc), *Trametes versicolor* (Tv), *Hypholoma fasciculare* (Hf), Whitestone (WS).

Procrustes analysis produced a correlation of 0.571 between bacterial and fungal communities (Procrustes sum of squares = 0.674, *P* = 0.001). Of the residual variance in bacterial−fungal occurrence (Fig. 3), 5.0% was explained by pre-coloniser identity, 15.7% by site and 11.8% by wood pH (adjusted *R^2^*, one-way ANOVAs). Soil pH had no explanatory power in fungal-bacterial co-occurrence.

### Drivers of bacterial richness

**Figure 4.**
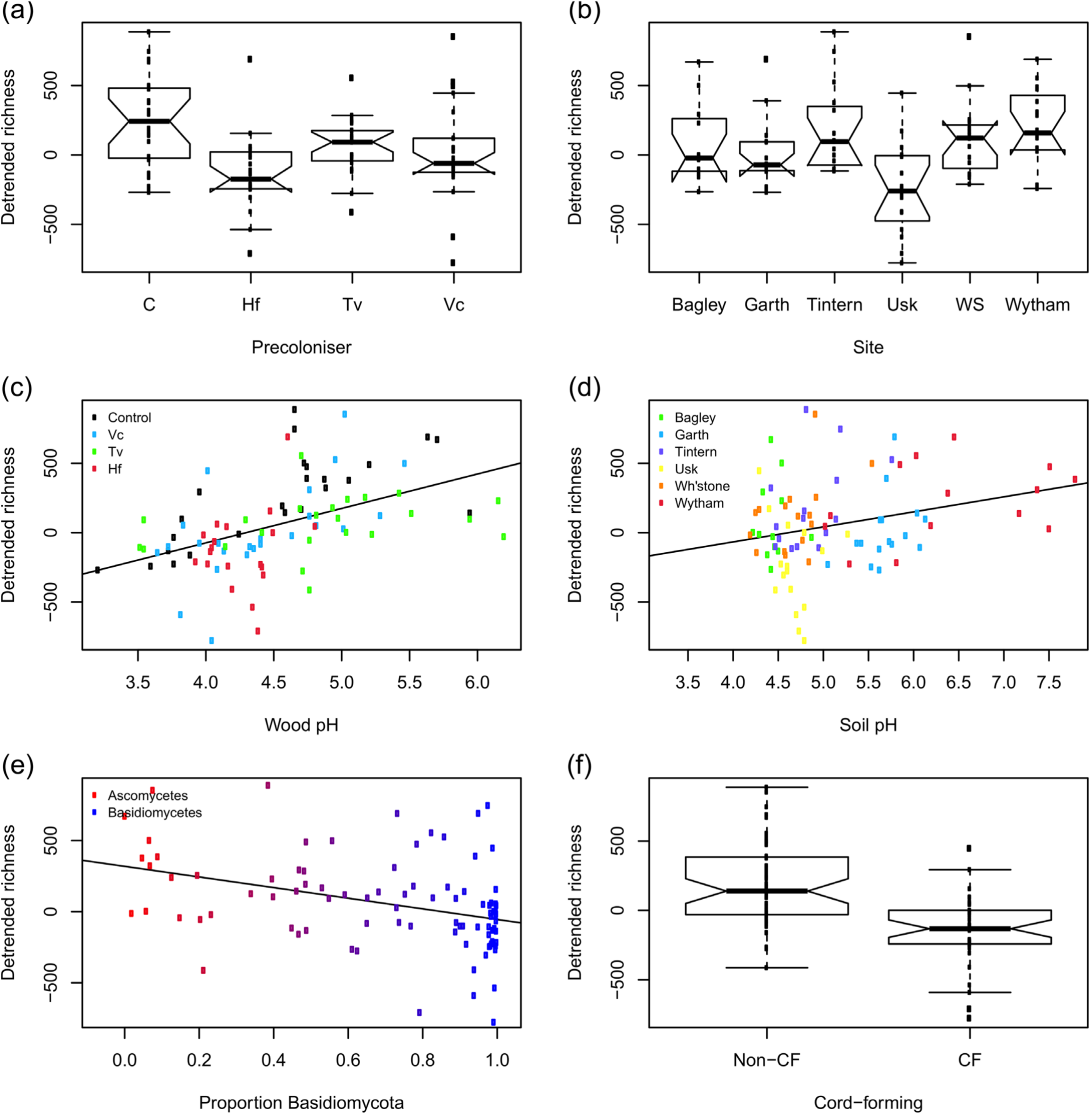
Overall bacterial OTU richness in fungus-colonised wood disks. Residuals from a model to correct for sequencing depth, broken down by (**a**) pre-coloniser identity; (**b**) site of origin; (**c**) wood pH (coloured by pre-coloniser); (**d**) soil pH (coloured by site); (**e**) relative proportions of ascomycete and basidiomycete reads and (**f**) whether the dominant fungal OTU belonged to a cord-forming genus. Individual data points are overlaid on boxplots. The *y*-scale relates to the number of OTUs relative to that predicted by sequencing depth alone, i.e. negative values denote samples with lower diversity than predicted by the null model. A simple regression line is overlaid on the scatterplots. Notches on boxplots represent 95% confidence intervals; where these extend beyond the quartiles, ‘hinges’ appear on the plot. Abbreviations are control (C), *Vulleminia comedens* (Vc), *Trametes versicolor* (Tv), *Hypholoma fasciculare* (Hf), Whitestone (WS), cord-former (CF).

Bacterial OTU richness was higher in the control than in pre-colonised disks (Fig. 4a; Table 3). The only site to show a marked difference in bacterial richness was Usk, which had on average fewer bacterial OTUs than the other sites (Fig. 4b). There was an upward trend in richness with increasing wood pH, but no effect of soil pH (Fig. 4c and d). Soil pH had a model coefficient of 1.0981, and wood pH of 1.395, i.e. a one-unit increase in soil pH corresponded to a 10% increase in OTU richness, compared to a 39% increase in richness for the same change in wood pH (Table 3). Therefore, when other factors were controlled for, wood pH was almost four times as important as soil pH in dictating bacterial richness. Bacterial richness was lower in basidiomycete-dominated disks compared to ascomycete-dominated disks (Fig. 4e), and decreased substantially when the dominant fungal OTU belonged to a cord-forming genus (Fig. 4f).

### Focus on taxa of interest

**Figure 5.**
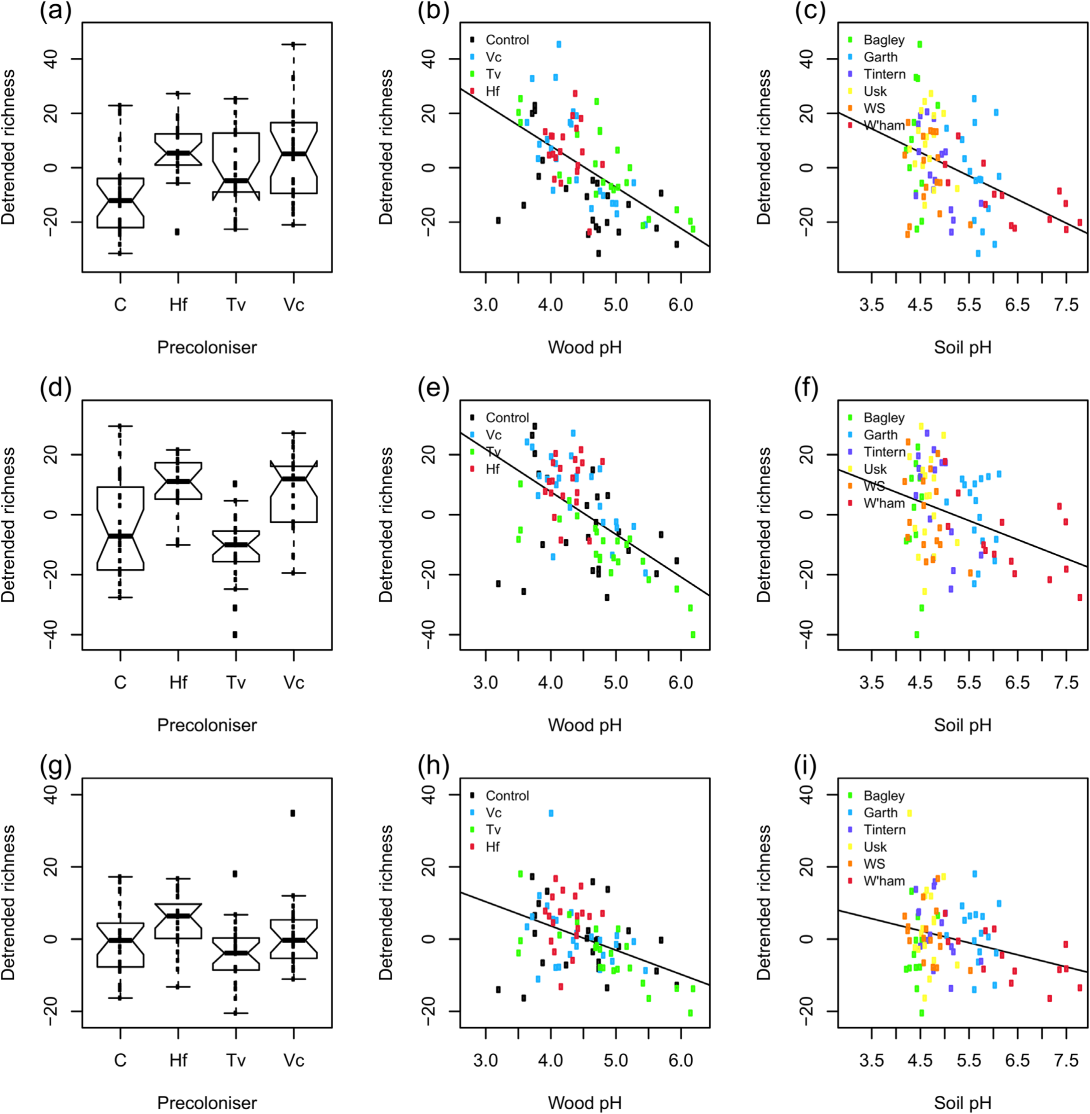
OTU richness for selected bacterial families in fungus-colonised wood disks. Residuals from models to correct for sequencing depth. (**a**) *Burkholderiaceae* broken down by pre-coloniser identity; (**b**) *Burkholderiaceae* broken down by wood pH (coloured by pre-coloniser); (**c**) *Burkholderiaceae* broken down by soil pH (coloured by site); (**d**)-(**f**) *Acidobacteriaceae* broken down by the same predictors and (**g**)-(**i**) *Acetobacteraceae* broken down by the same predictors. The *y*-scale relates to the number of OTUs relative to that predicted by sequencing depth alone, i.e. negative values denote samples with lower diversity than predicted by the null model. A simple regression line is overlaid on the scatterplots. Notches on boxplots represent 95% confidence intervals; where these extend beyond the quartiles, ‘hinges’ appear on the plot. Abbreviations are control (C), *Vulleminia comedens* (Vc), *Trametes versicolor* (Tv), *Hypholoma fasciculare* (Hf).

**Figure 6.**
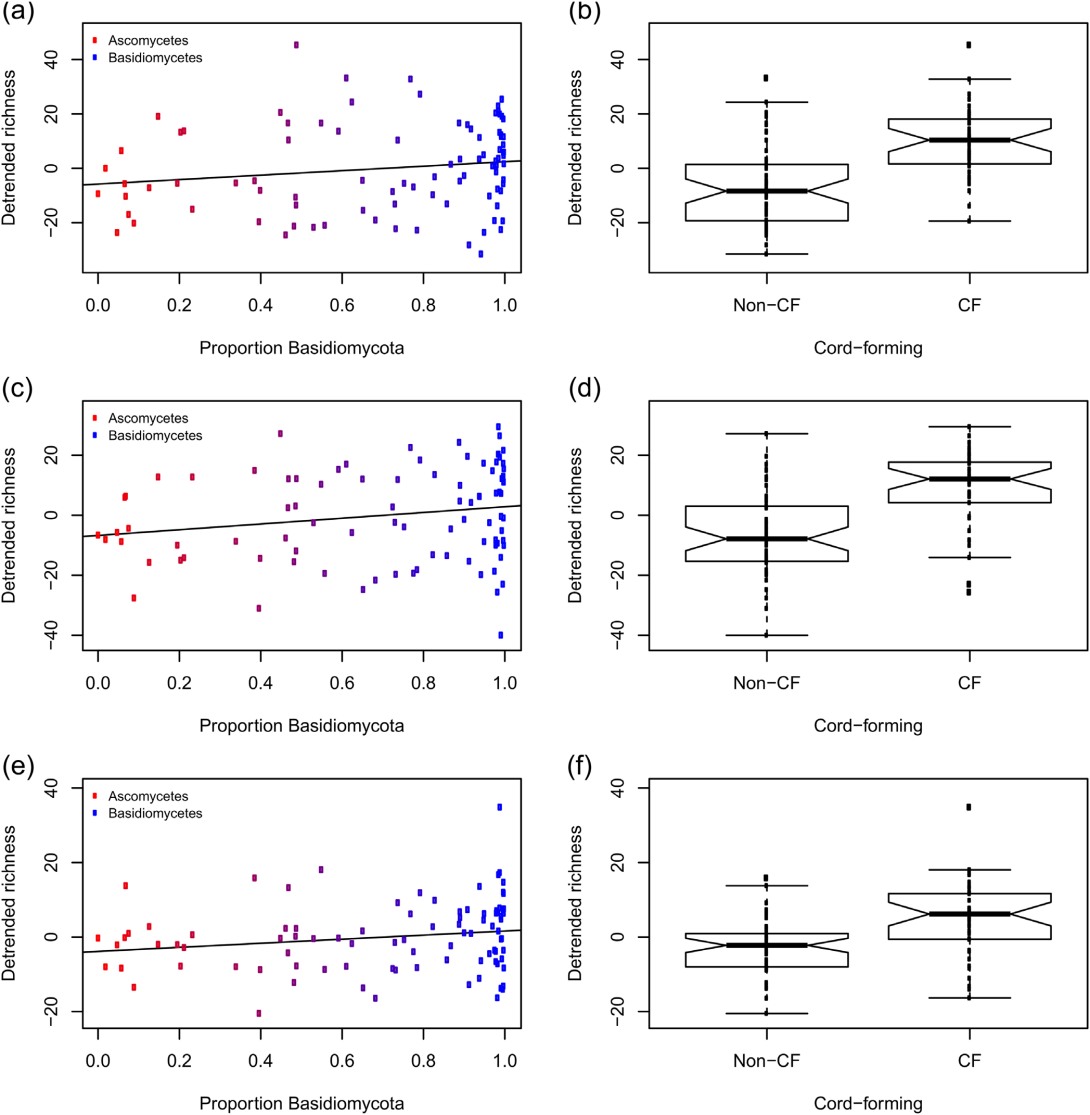
OTU richness for selected bacterial families in fungus-colonised wood disks in relation to the fungi present. Residuals from models to correct for sequencing depth (the *y*-scale is therefore arbitrary). (**a**) *Burkholderiaceae* broken down by the relative proportions of ascomycete and basidiomycete reads; (**b**) *Burkholderiaceae* broken down by whether the dominant fungal OTU belonged to a cord-forming genus; (**c**)-(**d**) *Acidobacteriaceae* broken down by the same predictors and (**d**)-(**f**) *Acetobacteraceae* broken down by the same predictors. A simple regression line is overlaid on the scatterplots. Notches on boxplots represent 95% confidence intervals. Abbreviations are control (C), *Vulleminia comedens* (Vc), *Trametes versicolor* (Tv), *Hypholoma fasciculare* (Hf), Whitestone (WS), cord-former (CF).

*Burkholderiaceae* showed an increased OTU richness and relative abundance in the pre-colonised samples compared to the control (Fig. 2; Fig. 5a). *Acidobacteriaceae* showed the same pattern, but only for *V. comedens* and *H. fasciculare* pre-colonised samples (Fig. 5d). *Acetobacteraceae* was notable for its increase only in *H. fasciculare* pre-colonised samples (Fig. 5g). Richness for each family varied slightly and idiosyncratically between sites (Fig. S6, Supporting Information). All three showed a negative trend with increasing wood and soil pH, although wood pH was twice as important as soil pH for *Acidobacteriaceae* and four times as important for *Burkholderiaceae* (Fig. 5; Table 3). The pre-coloniser relationships for each family held true even when pH was statistically controlled for (Table 3). None of these three bacterial families showed any trend based on the relative proportion of basidiomycetes in the disk, but all had higher richness when the dominant fungal OTU was a cord-former (Fig. 6).

## DISCUSSION

This is the first field study to examine fungus-bacteria associations in wood whilst experimentally manipulating fungal colonisers. Whilst fungi induce radical changes in bacterial community composition in wood microcosms (Folman *et al*. 2008; Hervé *et al*. 2014), the present study provided the first evidence of a causative association in the field. It revealed that the bacterial community is dependent on the ecological strategy of the dominant fungus, with competitive secondary colonisers reducing bacterial diversity and driving community shifts. This controlling effect of the dominant fungus was a more important determinant than either resource history or geographical location. The clear effect of wood pH on bacterial richness at the community and family level hints that pH manipulation may be a key means by which fungi exert their influence.

Forest soils are dominated by *Acidobacteria*, *Actinobacteria*, *Proteobacteria* and *Bacteroidetes* (Lladó, López-Mondéjar and Baldrian 2017). The relative dominance of *Proteobacteria* and *Acidobacteria* in the present study is consistent with previous studies in wood (Johnston, Boddy and Weightman 2016; Rinta-Kanto *et al*. 2016). *Firmicutes* were poorly represented, particularly in the fungal-pre-colonised samples, perhaps because they are more associated with mineral rather than organic soil horizons (Lladó, López-Mondéjar and Baldrian 2017). However, Hoppe *et al*. (2015) and Tláskal *et al*. (2017) found both *Firmicutes* and *Actinobacteria* were noticeable components of wood-inhabiting taxa. The low abundance of *Actinobacteria* in the present study may be related to their preference for higher-pH environments (Lladó, López-Mondéjar and Baldrian 2017). Among the *Bacteroidetes* present, *Chitinophagaceae* was a major component: given the abundance of chitin in fungal cell walls, this hints at bacterial predation or decomposition of fungal biomass.

### Fungal community composition is more important than resource history

The fungal and bacterial communities within the wood explained nearly 60% of the covariance between them. Significant co-occurrence patterns between fungi and bacteria have previously been observed in decomposing wood (Hoppe *et al*. 2014; Rinta-Kanto *et al*. 2016). Of the three possible explanations (fungi dictate bacteria; bacteria dictate fungi; both are dictated by the same environmental factors), all are likely to operate to a greater or lesser degree. This study addressed the first by manipulating the fungus initially present. *H. fasciculare* remained in the disks across the whole study period, so correlation with the bacterial community could only be driven by the fungus (as it was the factor under experimental control). By retaining its territory for the whole year, *H. fasciculare* also had the longest opportunity to select bacteria. The results confirmed that bacterial selection by *H. fasciculare* occurs not only in the lab (Folman *et al*. 2008; de Boer *et al*. 2010) but also in the field. Other pre-coloniser fungi were competitively replaced over the course of the experiment, and the bacterial communities for these treatments could not be separated from each other or the control disks. This indicates that it is the fungi currently present that shape the bacterial community, rather than the resource history. This nonetheless leaves room for a more subtle effect of previous colonisers, as they can influence the path of subsequent succession via priority effects (Hiscox *et al*. 2015, 2016).

One possibility is that *only* extremely combative fungi have the capacity to determine the bacterial community; this cannot be addressed in the present study, as the only pre-coloniser to retain its territory was the cord-forming *H. fasciculare*. Subsequent work (Johnston *et al*., in prep.) carried out over a shorter time span, addresses this problem by including wood where less combative pre-colonisers are still present at the time of collection.

### Fungal succession simplifies the bacterial community

All pre-coloniser treatments reduced bacterial OTU richness relative to the control: the later the successional position of the pre-coloniser, the greater the reduction in richness. It is important to note that after a year in the field the control disks were completely colonised by fungi, but at an earlier stage of fungal community development than the pre-colonised disks. Wood-decay fungi have been previously observed to reduce the number and diversity of bacteria in their resource (Folman *et al*. 2008). This simplification of the overall bacterial community occurred concurrently with enrichment of ‘fungus-tolerant’ bacteria such as *Burkholderiaceae*. Surveys of naturally decaying wood have found that bacterial richness and abundance increased with decay stage (Sun *et al*. 2014; Hoppe *et al*. 2015; Rinta-Kanto *et al*. 2016; Kielak *et al*. 2016b), although Tláskal *et al*. (2017) found no relationship between bacterial diversity and wood decay stage. Some of the above studies may have included wood at a very late stage of decay, when the highly competitive fungi have been replaced by stress-tolerant species (Boddy and Hiscox 2016). The relationship between decay stage and bacterial diversity is likely to be governed by an interplay of factors. On the one hand, progressively more competitive fungi may be expected as decay proceeds; pH decreases over the course of decay (Tláskal *et al*. 2017), which may be expected to negatively affect bacterial diversity. On the other hand, richness may increase due to increased water content, which tends to increase with decay (Hoppe *et al*. 2015); and nitrogen content also tends to increase with decay and is a strong predictor of bacterial abundance (Tláskal *et al*. 2017).

### Fungal ecology is more important than identity

The clearest separation between bacterial communities was driven by the ecological strategy of the dominant fungus. The ability to form mycelial cords is often associated with high competitive ability and a late secondary position in the successional hierarchy (Boddy 1993). Therefore, it is unsurprising that cord-forming fungi are adept at manipulating the bacterial community. More surprising is that this trait appears to be more important than the identity of the fungus concerned. There was no clear effect of fungal taxonomy on bacterial community composition. It has been suggested that wood-inhabiting bacteria respond to abiotic changes in wood (proximate cause) rather than fungi directly (ultimate cause) (Kielak *et al*. 2016b). The cord-formers had a greater chance to produce a discernible effect, because they generally occupied more of the disk and so had a greater influence over the sampling unit. This territory effect was not in itself sufficient to explain the separation in bacterial communities. However, it underlines the importance of single-species fungal dominance within a woody resource.

### pH is an important means of fungal resource control

Wood pH was an important determinant of bacterial richness, but was itself heavily influenced by the identity of the dominant fungus in the wood. This supports the idea that pH is an important means by which fungi control the wood environment, and specifically its bacterial community (de Boer *et al*. 2010). Counterintuitively, there was a negative relationship between PAM values and wood pH, suggesting that at low pH there is less concordance between bacterial and fungal communities. This is likely due to the influence of fungal richness, which negatively correlated with PAM: the disks containing the most competitive fungi tended to have lowest pH, lowest fungal and bacterial richness and therefore fewer OTUs to be correlated.

### Site is a less important determinant than fungal influences

Whilst sites did not show a clear clustering on the ordination plot, site nonetheless showed significant influence as a predictor. This may have been mediated by an altered fungal community between sites, leading to an altered pattern of succession (Hiscox *et al*. 2016). Support for this explanation comes from the clear separation between pre-coloniser treatments at Wytham, where Hiscox *et al*. (2016) found the most distinct fungal successor communities following each pre-coloniser. The Usk site showed differences to the other sites in its tendency to form a cluster in the ordination, and in its markedly lower bacterial richness. This may be due to the localised dominance of *Megacollybia platyphylla*, a highly competitive cord-forming basidiomycete, leaving its ‘signature’ on the bacterial community at the whole site. Another indication that the inter-kingdom relationship may be influenced by location is that site explained 16% of the residual variation in fungal−bacterial community correlation (although confidence intervals overlapped for all sites).

### PATTERNS IN TAXA

The three bacterial families selected for further exploration showed responses different to and often opposing the behaviour of the community as a whole, underlining the value of exploring individual taxa (Warton 2008). Of particular note is that all three decreased in richness with increasing pH; this is contrary to the usual pattern for soil bacteria, which are competitively disadvantaged compared to fungi at low pH (Rousk, Brookes and Bååth 2010), and indicates that these taxa are adapted both to fungal presence and to environments more amenable to fungal growth. It is possible that pH operates a two-stage filter on bacterial colonisation of wood: soil pH constrains the pool of colonists available to enter the resource, and wood pH constrains which of those are then capable of colonising the resource. This was not visible at the whole-community level, but did apply for these families.

*Burkholderiaceae* are outstanding among bacteria for their ability to form fungal associations (Johnston, Boddy and Weightman 2016), and in the pre-colonised wood they were markedly and consistently higher in richness and relative abundance compared to the controls. This affinity for fungal co-occurrence may be mediated partially by tolerance for low pH environments (Stopnisek *et al*. 2015); within soil, *Burkholderia* are most plentiful in slightly acidic environments around pH 5–6, but are still abundant at pH 3–4, more similar to wood decay environments (Stopnisek *et al*. 2014). *Acidobacteriaceae* are a relatively newly described and underexplored group of heterotrophic soil bacteria (Kielak *et al*. 2016a). The present study agrees with the limited pre-existing knowledge of this family, in that they show an affinity for low pH, low nutrient environments. At least some forest soil *Acidobacteria* have the capacity to metabolise chitin and the cellulose breakdown product, cellobiose (Lladó *et al*. 2015). It has been suggested that members of the phylum *Acidobacteria* are K-strategists (Kielak *et al*. 2016a), which may make them well-suited to this low-nutrient environment. *Acetobacteraceae* are also known for their acid tolerance and ability to metabolise a range of low-molecular weight carbon sources (Mamlouk and Gullo 2013). Intriguingly, given the low nitrogen content of wood, this family includes some diazotrophs (Reis and Teixeira 2015).

## CONCLUSIONS

Overall, this study underlines the importance of wood-decay fungi in controlling the dead-wood environment. In territory held by a highly competitive fungus, the bacterial community shifts towards acid-tolerant, metabolically versatile taxa adapted to the fungal environment. This study demonstrates for the first time that fungi drive bacterial community composition in the field. This relationship is particularly pronounced when the dominant fungus is a cord-former. Several bacterial families, notably *Burkholderiaceae*, show a marked positive association with fungal-colonised wood.

## Supplementary Material

Supplemental Files
