# Supplementary material for: Highly competitive fungi manipulate bacterial communities in decomposing beech wood (Fagus sylvatica)
Source: FEMS Microbiol Ecol. 2018 Nov 29;95(2):fiy225. doi: 10.1093/femsec/fiy225 (PMC6301287; doi:10.1093/femsec/fiy225)
Supplement: Supplemental Files [file fiy225_supplemental_files.zip › Johnston-et-al_bioinf.pdf]

###Reanalysis of multisite sequences, 2016

#This file records a list of steps used, and the commands that produced them. It also acts as a log of what each sub-directory contains.

#Working directory is 2016Sarah-reanalysis

#Outputs of each step saved in a new numbered directory

#NB This document does not include any optomisation of filtering parameters, extensive examination of fastqc reports etc.

#Raw demultiplexed sequences are in the folder ../Petr_demultiplexed/all_samples

#The folder also contains a file called barcode_lengths.csv which contains 3 fields: sample number, F barcode length, R barcode length.

#Remove the barcodes from each sequence and save output files to directory '1-barcodes_removed'

> cat /home/GROUP-sabajw2/project/multisite/Petr_demultiplexed/all_samples/barcode_lengths.csv | while read a b c; do cat /home/GROUP-sabajw2/project/multisite/Petr_demultiplexed/all_samples/${a}.fastq | fastx_trimmer -f ${b} | fastx_trimmer -t ${c} > 1-barcodes_removed/${a}_trimmed.fastq; done

#Filter sequences to only keep those with complete, correct primer sites and remove the primers using trim_primers.sh - save output files to directory '2-primers_removed'

#Time it to see how long it takes - 100m7.259s

> time ../trim_primersv2.sh 1-barcodes_removed 2-primers_removed

#Use usearch to remove sequences that are too short or contain a 2 max expected errors, saving output reports to file.

#Ouput files in directory 3-usearch_filtered

#Downloaded USEARCH v9.0.2132, chmod +x to make it executable, and appended directory to PATH

> export PATH=/home/GROUP-sabajw2:$PATH

#Time 4m54.086s

> time for i in {1..192}; do echo 2-primers_removed/primer_removed_sample.${i}_trimmed.fastq; usearch9.0.2132 -fastq_filter 2-primers_removed/primer_removed_sample.${i}_trimmed.fastq -fastq_minlen 400 -fastq_maxee 2 -fastaout 3-usearch_filtered/usearch_fasta/sample.${i}filtered.fasta -fastqout 3-usearch_filtered/usearch_fastq/sample.${i}_filtered.fastq -relabel ${i}; done > usearch_output.txt 2>&1

#Run fastqc on them all to get quality reports

#Output files in directory 3-usearch_filtered/fastqc_reports

> ls 3-usearch_filtered/usearch_fastq | while read f; do fastqc -o 3-usearch_filtered/fastqc_reports 3-usearch_filtered/usearch_fastq/$f; done

#Checked through a selection of fastqc reports: 1, 10, then every 10th sample until 190. Then went through the reports for samples that were flagged as problematic previously - 145 (U C), 178 (Wy Hf), 192 (Sarah kit control). Base quality acceptable on all of them, but 'problematic' samples had low read numbers. Same strange length distribution - wonder if it could be linked to fungal/eukaryote amplification?

#Split multisite fastas off from other samples in the same run

> for i in {98..192}; do mv sample.${i}filtered.fasta ./multisite_fastas; done

#Combine filtered fasta files ready for QIMME (done in QIIME version 1.9.1+dfsg-1biolinux4)

#Output file is directory 4-labelled_fastas and subdir multisite

#Map is multisite_map.tsv

#Time 1m54.641s

> time add_qiime_labels.py -i 3-usearch_filtered/usearch_fasta/multisite_fastas -m multisite_map.tsv -c InputFileName -o 4-labelled_fastas/multisite

#Chimera checking in QIIME

#Output files in directory 5-chimera_checking subdir multisite

#Time 10m12.631s for multisite

> time identify_chimeric_seqs.py -m usearch61 -i /home/GROUP-
sabajw2/project/multisite/2016Sarah-reanalysis/4-labelled_fastas/multisite/combined_seqs.fna -r
/home/GROUP-sabajw2/project/multisite/gg_otus_4feb2011/rep_set/gg_97_otus_4feb2011.fasta -
o /home/GROUP-sabajw2/project/multisite/2016Sarah-reanalysis/5-chimera_checking/multisite

#Remove chimeras from input files in QIIME

#Output files in directory 5-chimera_checking subdir multisite

#Time 0m56.925s for multisite

> time filter_fasta.py -f /home/GROUP-sabajw2/project/multisite/2016Sarah-reanalysis/4-
labelled_fastas/multisite/combined_seqs.fna -o /home/GROUP-
sabajw2/project/multisite/2016Sarah-reanalysis/5-
chimera_checking/multisite/multisite_seqs_chimeras_filtered.fna -s /home/GROUP-
sabajw2/project/multisite/2016Sarah-reanalysis/5-chimera_checking/multisite/chimeras.txt -n

#Assign multisite sequences to OTUs at 97% sequence similarity, using QIIME open reference method and the greengenes bacterial database

#OTU picking on multiple (4) threads, labelling de novo OTUs with 'SJnew'

#Output into directory 6-OTUs/multisite/gg

#Time 15m18.620s

> time pick_open_reference_otus.py -i /home/GROUP-sabajw2/project/multisite/2016Sarah-
reanalysis/5-chimera_checking/multisite/multisite_seqs_chimeras_filtered.fna -o //home/GROUP-
sabajw2/project/multisite/2016Sarah-reanalysis/6-OTUs/multisite/gg -r /home/GROUP-
sabajw2/project/multisite/gg_otus_4feb2011/rep_set/gg_97_otus_4feb2011.fasta -n SJnew -a -O 4

#Assign multisite sequences to OTUs at 97% sequence similarity, using QIIME open reference method and the SILVA database

#OTU picking on multiple (4) threads, labelling de novo OTUs with 'SJnew'

#Output into directory 6-OTUs/multisite/SILVA

#Time

> time pick_open_reference_otus.py -i /home/GROUP-sabajw2/project/multisite/2016Sarah-
reanalysis/5-chimera_checking/multisite/multisite_seqs_chimeras_filtered.fna -o /home/GROUP-
sabajw2/project/multisite/2016Sarah-reanalysis/6-OTUs/multisite/SILVA -r /home/GROUP-
sabajw2/project/multisite/SILVA/Silva119_release_aligned_rep_files/97/Silva_119_rep_set97_align
ed.fna -n SJnew -a -O 4

#Convert .biom OTU tables to .tsv

> biom convert -i /home/GROUP-sabajw2/project/multisite/2016Sarah-reanalysis/6-OTUs/multisite/gg/otu_table_mc2.biom -o /home/GROUP-sabajw2/project/multisite/2016Sarah-reanalysis/6-OTUs/multisite/gg/multisite_gg_otu_table.tsv --to-tsv

> biom convert -i /home/GROUP-sabajw2/project/multisite/2016Sarah-reanalysis/6-OTUs/multisite/SILVA/otu_table_mc2.biom -o /home/GROUP-sabajw2/project/multisite/2016Sarah-reanalysis/6-OTUs/multisite/SILVA/multisite_silva_otu_table.tsv --to-tsv

#Add in metadata to the biom files; this command adds both sample and observation metadata, and should specify that the taxonomy is semicolon-delimited

#Mark new files as 'metatax' to denote that they include metadata and taxonomy

#Need to add a header line to each taxonomy file

> sed -i '1s/^/#SampleID\tTaxonomy\tConfidence\n/' 6-OTUs/multisite/gg/uclust_assigned_taxonomy/rep_set_tax_assignments.txt

> sed -i '1s/^/#SampleID\tTaxonomy\tConfidence\n/' 6-OTUs/multisite/SILVA/uclust_assigned_taxonomy/rep_set_tax_assignments.txt

> biom add-metadata -i 6-OTUs/multisite/gg/otu_table_mc2.biom -o 6-OTUs/multisite/gg/otu_table_metatax.biom --observation-metadata-fp 6-OTUs/multisite/gg/uclust_assigned_taxonomy/rep_set_tax_assignments.txt --sample-metadata-fp multisite_map.txt --sc-separated taxonomy

> biom add-metadata -i 6-OTUs/multisite/SILVA/otu_table_mc2.biom -o 6-OTUs/multisite/SILVA/otu_table_metatax.biom --observation-metadata-fp 6-OTUs/multisite/SILVA/uclust_assigned_taxonomy/rep_set_tax_assignments.txt --sample-metadata-fp multisite_map.tsv --sc-separated taxonomy

#Used custom script add_taxonomy_to_otu_table.sh to add taxonomy to the tsv. Renamed files to multisite_gg_OTU_table_with_taxonomy.tsv and multisite_silva_OTU_table_with_taxonomy.tsv

#Compare SILVA and greengenes results for multisite.

#Moved into 6-OTUs/multisite for this part.

```
> cat gg/multisite_gg_OTU_table_with_taxonomy.tsv | cut -f 97 | sort | uniq

Bacteria

Kingdom

Unassigned

> cat SILVA/multisite_silva_OTU_table_with_taxonomy.tsv | cut -f 97 | sort | uniq

Bacteria

Kingdom

Unassigned

#So SILVA doesn't return any fungal records either

> cat gg/multisite_gg_OTU_table_with_taxonomy.tsv | cut -f 97 | grep -c 'Unassigned'

372

> cat SILVA/multisite_silva_OTU_table_with_taxonomy.tsv | cut -f 97 | grep -c 'Unassigned'

375

#Very similar number of unassigned OTUs at the Kingdom level

> cat gg/multisite_gg_OTU_table_with_taxonomy.tsv | cut -f 98 | sort | uniq -c

    373

    374  Acidobacteria

    379  Actinobacteria

     12  Armatimonadetes

   1061  Bacteroidetes

      1  BHI80-139

    222  Chlamydiae

      4  Chlorobi

     36  Chloroflexi

     59  Cyanobacteria

      3  Elusimicrobia

     11  FBP
```

```
      2  FCPU426
      3  Fibrobacteres
    183  Firmicutes
     26  Gemmatimonadetes
      1  GN02
      1  Lentisphaerae
     41  OD1
      1 Phylum
    407  Planctomycetes
   3717  Proteobacteria
      9  Spirochaetes
      1  SR1
      4  Tenericutes
      1  [Thermi]
     64  TM6
    152  TM7
    397  Verrucomicrobia
     29  WPS-2
> cat SILVA/multisite_silva_OTU_table_with_taxonomy.tsv | cut -f 98 | sort | uniq -c
    376
    372  Acidobacteria
    247  Actinobacteria
     11  Armatimonadetes
    946  Bacteroidetes
      1  BHI80-139
    223  Chlamydiae
      4  Chlorobi
```

34 Chloroflexi

57 Cyanobacteria

3 Elusimicrobia

11 FBP

2 FCPU426

3 Fibrobacteres

138 Firmicutes

25 Gemmatimonadetes

1 GN02

1 Lentisphaerae

41 OD1

1 Phylum

385 Planctomycetes

3013 Proteobacteria

5 Spirochaetes

1 SR1

4 Tenericutes

1 [Thermi]

63 TM6

143 TM7

344 Verrucomicrobia

22 WPS-2

#In light of the similarities and lack of fungal hits, it seems reasonable to go with greengenes as the database of choice.

#Diversity analyses

#Output files in directory 7-diversity/multisite

#Create a summary of the otu table to decide on rarefaction depth

biom summarize-table -i /home/GROUP-sabajw2/project/multisite/2016Sarah-reanalysis/6-OTUs/multisite/gg/otu_table_metatax.biom -o /home/GROUP-sabajw2/project/multisite/2016Sarah-reanalysis/6-OTUs/multisite/gg/multisite_gg_otu_table_summary.txt

#QIIME core diversity analysis

> core_diversity_analyses.py -i /home/GROUP-sabajw2/project/multisite/2016Sarah-reanalysis/6-OTUs/multisite/gg/otu_table_metatax.biom -o /home/GROUP-sabajw2/project/multisite/2016Sarah-reanalysis/7-diversity/multisite/core_div -m /home/GROUP-sabajw2/project/multisite/2016Sarah-reanalysis/multisite_map.tsv -e 866 -t /home/GROUP-sabajw2/project/multisite/2016Sarah-reanalysis/6-OTUs/multisite/gg/rep_set.tre -a -O 4

#Create a biom file that only has the experimental samples in it, so only these will be plotted

#QIIME

> filter_samples_from_otu_table.py -i 6-OTUs/multisite/gg/otu_table_metatax.biom -o 6-OTUs/multisite/gg/otu_table_exp_only.biom -m multisite_map.tsv -s 'Precoloniser:*,!N/A'

#Remove sample T10, which is of uncertain identity

> filter_samples_from_otu_table.py -i 6-OTUs/multisite/gg/otu_table_exp_only.biom -o 6-OTUs/multisite/gg/otu_table_minusT10.biom -m multisite_map.tsv -s 'SampleID:*,!T10'

> biom convert -i 6-OTUs/multisite/gg/otu_table_minusT10.biom -o 6-OTUs/multisite/gg/otu_table_minusT10_json.biom --table-type="OTU table" --to-json

#Create a map of only the experimental samples and excluding T10

> cat multisite_map.tsv | awk '$7 != "N/A" {print $0}' | awk '$1 != "T10" {print $0}' > multisite_map_exp_minusT10.tsv

#Run the taxa summary on the filtered biom, grouping samples by precoloniser

> summarize_taxa_through_plots.py -o 7-diversity/multisite/plot_summary_precol_minusT10 -i 6-OTUs/multisite/gg/otu_table_minusT10_json.biom -m multisite_map_exp_minusT10.tsv -c Precoloniser

#Counted number of sequences to pass quality filtering (excluding kit control sequences)

> cat 5-chimera_checking/multisite/multisite_seqs_

chimeras_filtered.fna | grep -v "Sarah.kit" | grep -c "^>"

2710316

#Checked and grepping "Sarah.kit" produced 296 results
