# Supplementary material for: Highly competitive fungi manipulate bacterial communities in decomposing beech wood (Fagus sylvatica)
Source: FEMS Microbiol Ecol. 2018 Nov 29;95(2):fiy225. doi: 10.1093/femsec/fiy225 (PMC6301287; doi:10.1093/femsec/fiy225)
Supplement: Supplemental Files [file fiy225_supplemental_files.zip › TableS1.docx]

Table S1. Sites used in the experiment. All sites were wooded, with *Fagus sylvatica* present. This table is adapted from Hiscox *et al.* (2016).

| Name | County | Grid reference  (lat., long.) | Tree species present | Predominant local soil type | Soil pH | Ground cover  % Species | |
| --- | --- | --- | --- | --- | --- | --- | --- |
| Gwaelod-y-garth | Cardiff | 51.535784,  -3.277605 | *F. sylvatica*, *Quercus sp*., *Ilex aquifolium* | Freely draining, slightly acid but base-rich soil | 4.70 | <5 | Fern, sedge, bramble |
| Usk | Monmouthshire | 51.672524,  -2.953690 | Predominantly *F. sylvatica*, some *Fraxinus excelsior*, *Castanea sativa* and *Acer psuedoplatanus* | Freely-draining, slightly acid, loamy soil | 4.68 | <5 | Woodrush, fern, ivy |
| Tintern | Monmouthshire | 51.711529,  -2.684246 | Predominantly *Quercus sp*, some *F. sylvatica* and *I. aquifolium* | Freely-draining, slightly acid, loamy soil | 5.67 | 95 | Bluebells, bramble, ferns, sedge |
| Whitestone | Monmouthshire | 51.725378,  -2.690553 | *F. sylvatica*, *Quercus sp*, *A. psuedoplatanus* | Freely-draining, slightly acid, loamy soil | 4.95 | 75 | Bluebells, ivy, ferns, bramble, ivy, wood anemone |
| Wytham | Oxfordshire | 51.768902,  -1.344022 | Predominantly *F. sylvatica*, some *Corylus avellana* | Shallow lime-rich soil over chalk or limestone | 6.58 | <5 | Sedge, moss |
| Bagley | Oxfordshire | 51.720134,  -1.266750 | *F. sylvatica*, *C. avellana*, *I. aquifolium* | Slowly permeable, seasonally wet, acid loamy and clayey soil | 4.52 | 25 | Fern, bramble, sedge |

Soil type information from Cranfield University 2015. *The Soils Guide*. Available: www.landis.org.uk. Cranfield University, UK. Last accessed 09/06/2015
