# Supplementary figures and images for: Highly competitive fungi manipulate bacterial communities in decomposing beech wood (Fagus sylvatica)
Source: FEMS Microbiol Ecol. 2018 Nov 29;95(2):fiy225. doi: 10.1093/femsec/fiy225 (PMC6301287; doi:10.1093/femsec/fiy225)

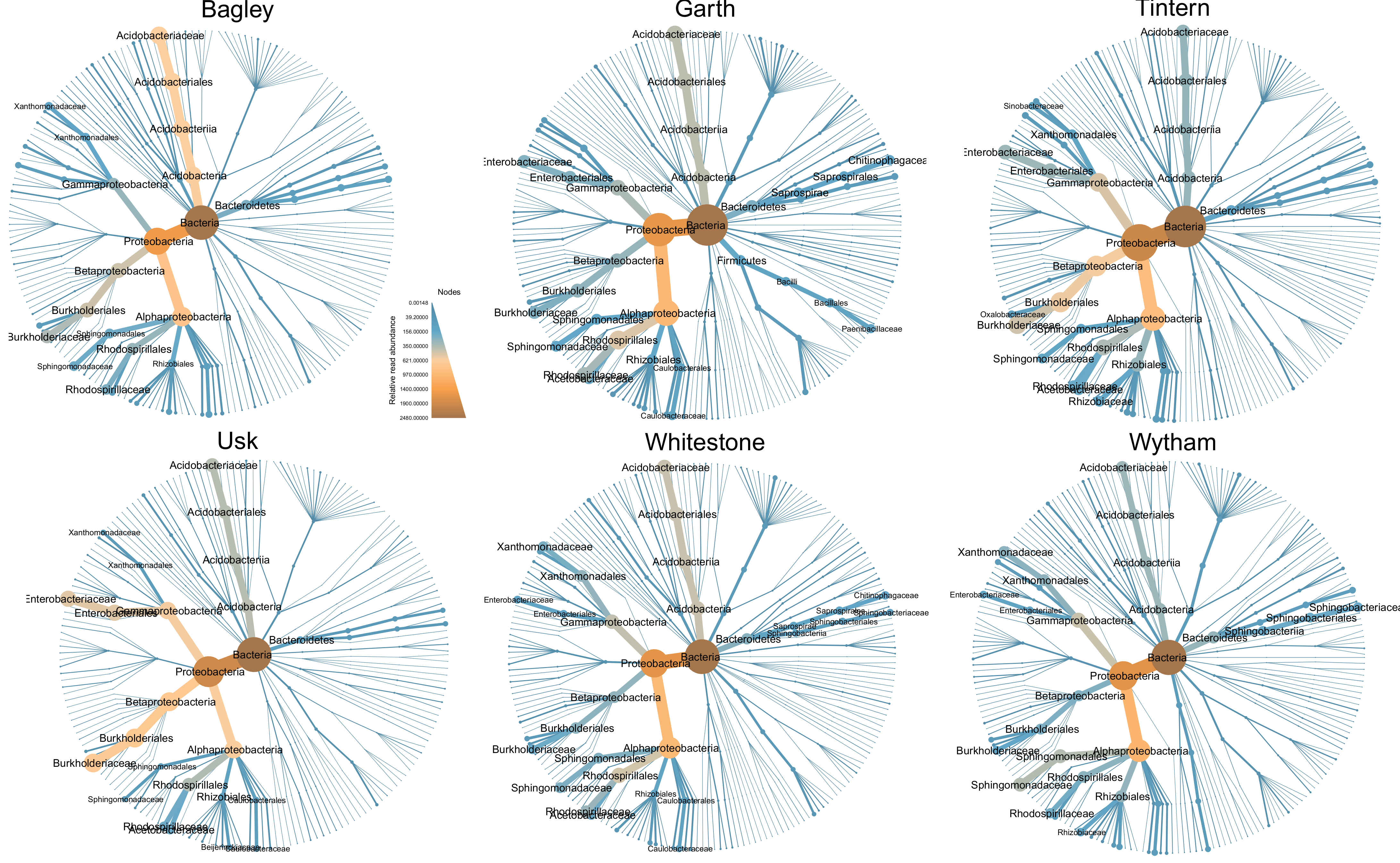

Supplement: Supplemental Files [file fiy225_supplemental_files.zip › FigS5.tiff]
